# Diversity of the non-targeted metabolomic data across various varieties of Cloves (*Syzygium* spp.)

**DOI:** 10.1016/j.dib.2024.111237

**Published:** 2024-12-16

**Authors:** Jakty Kusuma, Analianasari, Anung Wahyudi, Muhammad Khalid Abdullah, Ahmad Zainul Hasan, Imam Asrowardi, Fitriani, Muhammad Tahir

**Affiliations:** a Politeknik Negeri Lampung, Indonesia; b Corpora Science Research Laboratory, Yogyakarta, 55223 Indonesia

**Keywords:** Cloves, Chromatography, Spices, Moluccas, Boactive compounds

## Abstract

Cloves (*Syzygium aromaticum*), a tree in the Myrtaceae family, are indigenous to the Maluku Islands in Indonesia and are widely utilized as a spice. Essential oils are commonly extracted from clove leaves, flower buds, and stalks. However, due to supply constraints, other clove species, notably *Syzygium obtusifolium*, are sometimes used as substitutes, leading to lower-grade essential oils. Here, we employed a non-targeted mass-spectrometry-based metabolomics approach to characterize the metabolic profiles of leaves from ten clove varieties, including *S. obtusifolium*. We identified and quantified 427 metabolites across various metabolic pathways. The metabolomics data for all samples are publicly available at the Figshare repository under 10.6084/m9.figshare.27212016. The data can be accessed directly at https://figshare.com/s/f4a40b7903b6a946b203*.*

Specifications Table

**Table:** 

Subject	Agronomy and Crop Science, Metabolomics, Food chemistry, Chemistry of natural products.
Specific subject area	Metabolomic study for spice plants.
Type of data	Metabolomic data.
Data collection	Field work was conducted to collect leaves samples of various cloves varieties. Samples were dried and prepared, and then were analyzed using LC-HRMS.
Data source location	Moluccas archipelago, Indonesia.
Data accessibility	Repository name: Figshare Data identification number: 10.6084/m9.figshare.27212016 Direct URL to data: https://figshare.com/s/f4a40b7903b6a946b203
Related research article	None

## 1 Value of the Data

• The data identifies and quantifies the types and concentrations of metabolites present in various varieties of clove leaves.
• This data provides, for the first time, a non-targeted metabolomic profile of various cloves leaf varieties.

## 2 Background

In Indonesia, the diverse varieties of cloves [2] are highly valued for their aromatic oils, which significantly influence market pricing. Each variety of clove offers a distinct aromatic quality, impacting its economic value. However, due to high demand, there are instances where farmers use non-aromatic species such as *Syzygium obtusifolium* [7] to supplement clove supplies. These are often mixed with aromatic varieties, complicating quality assessments, which traditionally rely solely on olfactory evaluation. This method, while intuitive, lacks consistency and fails to provide a measurable standard for grading clove quality. To address this issue, our study has developed a comprehensive metabolomics dataset, focusing on the metabolite profiles of nine aromatic clove varieties and one non-aromatic variety across the Moluccas archipelago. This dataset aims to establish a more objective and scalable method for grading clove quality.

## 3 Data Description

**Fig. 1:**
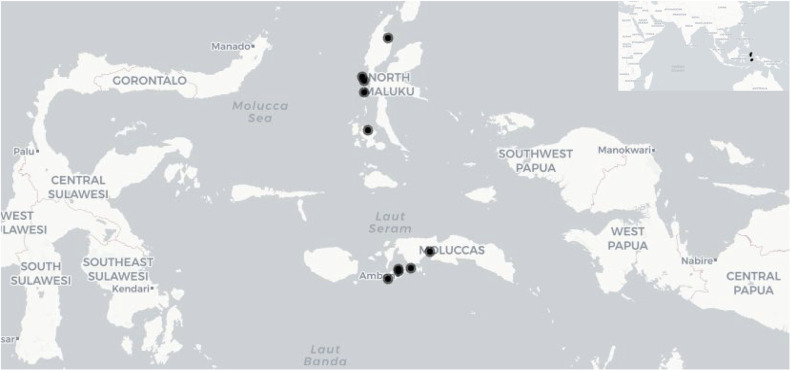
Sampling location of samples used in this study are indicated in black dot. *inset*: Moluccas archipelago is situated in the eastern part of the Indo-Malaya archipelago.

**Fig. 2:**
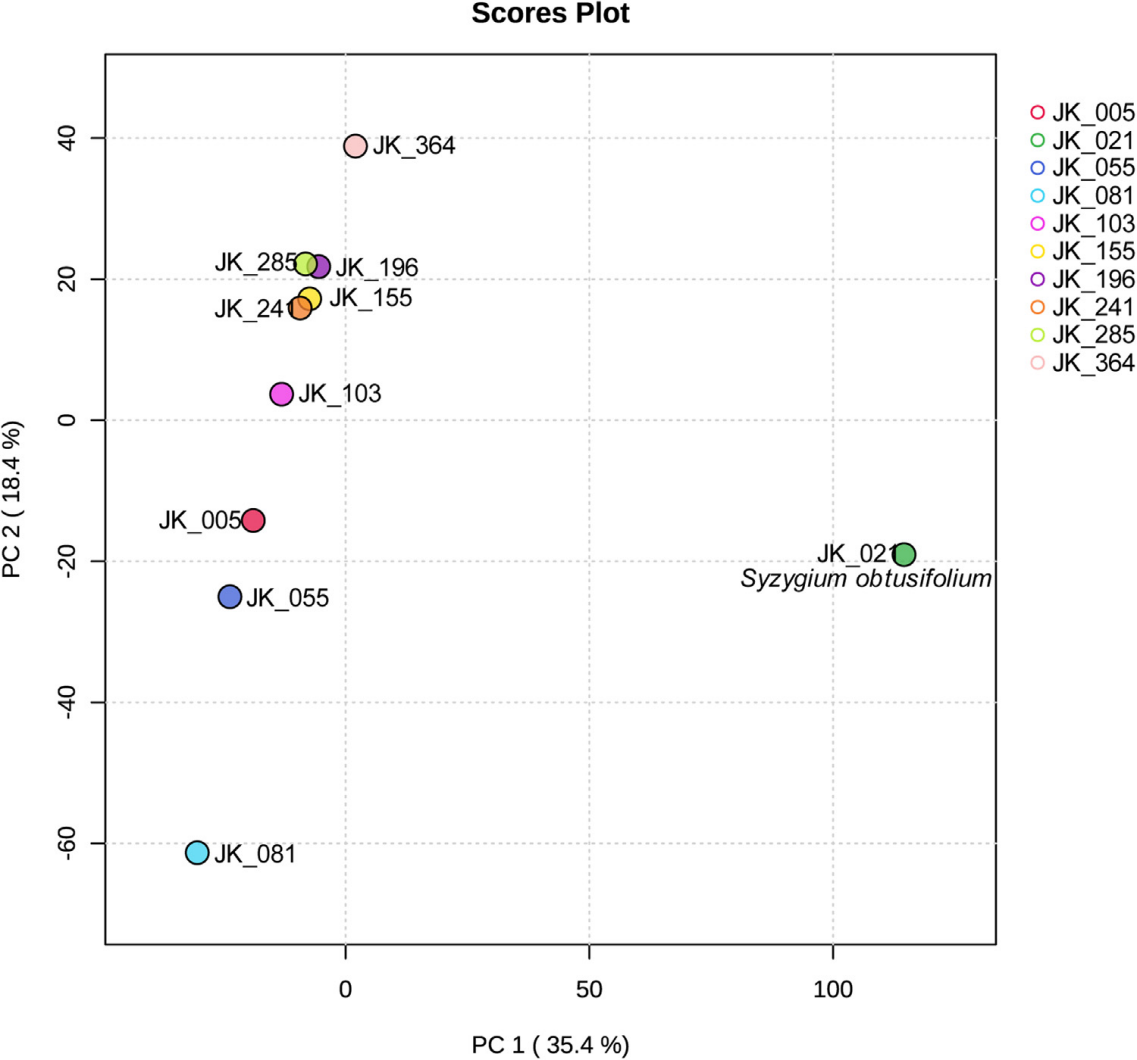
Principal component analysis (PCA) of ten samples.

**Fig. 3:**
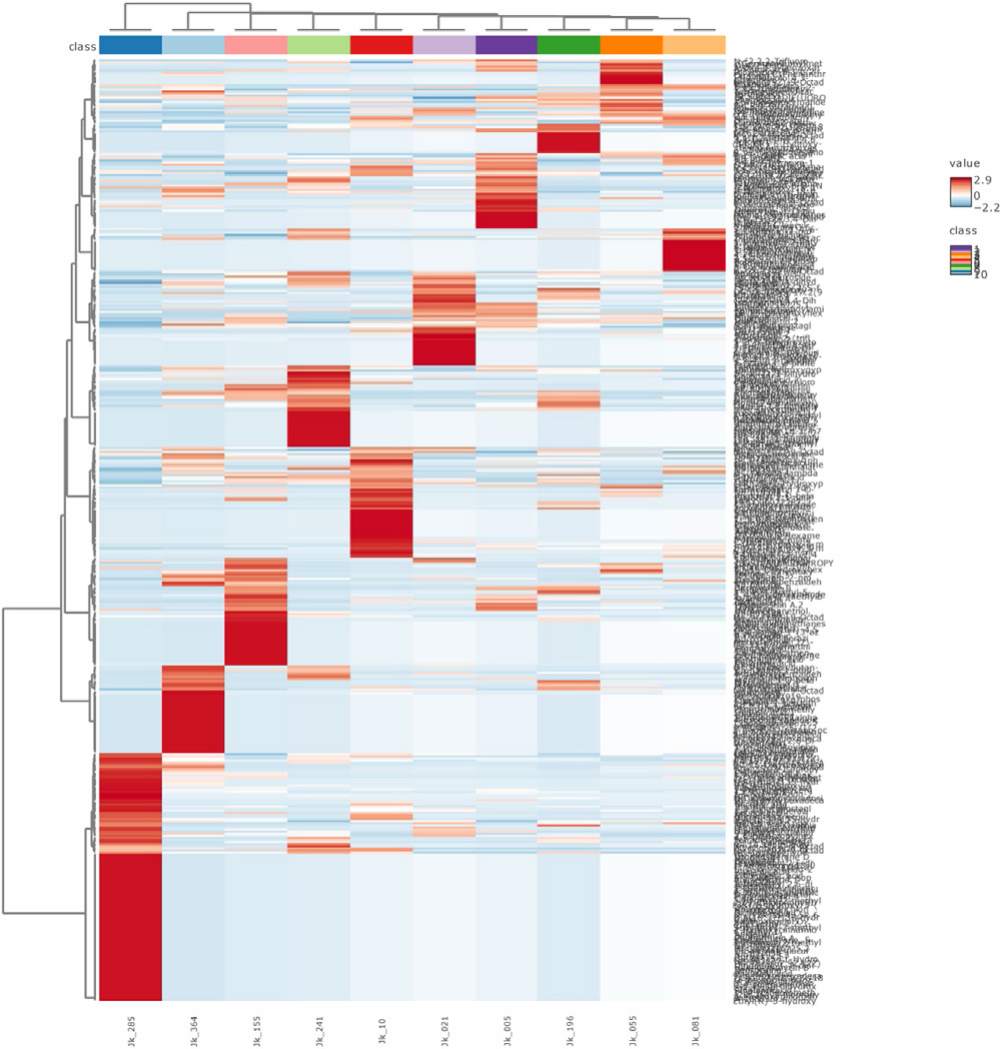
Clustering result shown as heatmap.

In this report, we present a detailed, non-targeted metabolomics dataset that includes nine varieties of cloves (*Syzygium aromaticum*) and one variety of *Syzygium obtusifolium* leaves, as catalogued in Table 1. This dataset is composed of samples gathered from various sites across the Moluccas archipelago in Indonesia, with their specific locations illustrated in Fig. 1. Metabolomics, as utilized here, facilitates the comprehensive identification and quantification of the full spectrum of metabolites present within a plant cell or tissue [5,6]. We generated 452 compound and the metabolomic data were analyzed using MetaboAnalyst ver. 6.0 [4]. Principal component analysis (PCA) showed 53.80 % of total variance in the metabolomic profiles of the genotypes (Fig. 2). The species *S. obtusifolium* was distinctly separated from the remainder of the genotypes by the first principal component (PC1), which explained 35.4.8 % of the variance. The heatmap illustrates the distinct metabolic profiles across varieties, highlighting variations in metabolomic profiles (Fig. 3). Raw data is available at https://github.com/jaktykusuma/cloves.

**Table 1:** Sampling overview of *Syzygium* species across the moluccas archipelago.

No	Samples	Species	Varieties	Localities	Island	Habitat	Latitude	Longitude
1	JK_005	*Syzygium aromaticum*	Tuni	Wakasihu	Ambon	Secondary forest	−3.77067	127.95171
2	JK_021	*Syzygium obtusifolium*	Hutan Raja	Mamala	Ambon	Forest	−3.55854	128.19442
3	JK_055	*Syzygium aromaticum*	Sikotok	Pelauw	Haruku	Forest	−3.53496	128.47607
4	JK_081	*Syzygium aromaticum*	Boiselang	Masohi	Seram	Secondary forest	−3.60623	128.18887
5	JK_103	*Syzygium aromaticum*	Damar	Waisala	Seram	Mountain side	−3.17118	128.90881
6	JK_155	*Syzygium aromaticum*	Afo	Marikurubu	Ternate	Hills	0.79140	127.36359
7	JK_196	*Syzygium aromaticum*	Red Zanzibar	Fobaharu	Tidore	Hills	0.69524	127.39693
8	JK_241	*Syzygium aromaticum*	Kampung	Tafaga	Moti	Hills	0.44835	127.39136
9	JK_285	*Syzygium aromaticum*	Sibela	Yaba	Bacan	Forest	−0.42360	127.48713
10	JK_364	*Syzygium aromaticum*	Red Zanzibar	Tobelo	Halmahera	Forest	1.67225	127.94862

## 4 Experimental Design, Materials and Methods

We collected leaves from ten different clove varieties in North and South Moluccas, Indonesia. We used Leaf Plastochron Index (LPI) with value of *LPI* between 0.54 to 0.80 (see [1,3]), with length 7 cm and width 3 cm. The leaves were geotagged and dried using silica gel for sample preparation.

Each sample were weighed 50 mg except for sample JK_285 (16.4 g) due to the limited availability. 1 mL HPLC grade methanol were then added to each sample and vortexed for 1 min. Afterwards, they were sonicated for 30 min at 25 °C. This step was followed with centrifugation at 1400 × *g* for 5 min. The supernatants were then filtrated through 0.22 um nylon filters into HPLC vials.

Non-targeted metabolomics analysis was performed according to the published method by Windarsih et al. [8] with slight modifications. Ultra high-performance liquid chromatography (UHPLC, Vanquish Horizon, Thermo Scientific™, Germany) connected to a high-resolution mass spectrometer (HRMS Orbitrap™ Exploris™ 240, Thermo Scientific™, Germany) was used for non-targeted metabolomics analysis. A reverse-phase chromatography using Accucore™ Phenyl Hexyl, 10 cm × 2.1 mm × 2.6 µm column. The mobile phase consisted of water (A) and acetonitrile (B) containing 0.1 % formic acid for each mobile phase. The analytes were subjected to a gradient elution technique as follows: 5 % B and increased gradually to 90 % in 16 min, held at 90 % for 4 min and back to 5 % B until 25 min for column re-equilibration. The samples were injected at 5 µL. During the analyte separation, the column temperature was maintained at 40 °C. The HRMS parameters i.e. sheath gas flow rate and auxiliary gas flow rate, were set at 35 arbitrary units (AU) and 7 AU, respectively. Heated electrospray ionization (H-ESI) was used in both positive and negative modes employing a spray voltage of 3.5 kV. Before the analysis, both H-ESI positive and H-ESI negative were calibrated using Pierce™ FlexMix™ Calibration Solution (Thermo Scientific™, USA). The temperature for capillary was set at 300 °C during the analysis. The compounds were scanned at mass ranges from 70 to 800 *m/z* using resolution of 90,000 FWHM and 22,500 FWHM for MS1 and MS2, respectively. The normalized collision energies (NCE) applied were 30, 50, and 70 for both positive and negative ionization mode.

## Limitations

While this study offers a comprehensive analysis of the diversity in clove plants, it focuses solely on the leaves, excluding the buds, which are also significant for their commercial and aromatic properties. Additionally, specific environmental conditions, which could significantly influence metabolomic profiles, were not meticulously measured. These factors may affect the generalizability of the findings to other parts of the clove plant and other growing conditions*.*

## Ethics Statement

The authors have read and follow the ethical requirements for publication in Data in Brief and confirming that the current work does not involve human subjects, animal experiments, or any data collected from social media platforms.

## CRediT authorship contribution statement

**Jakty Kusuma:** Conceptualization, Methodology, Writing – original draft, Funding acquisition. **Analianasari:** Validation, Investigation. **Anung Wahyudi:** Data curation, Writing – review & editing. **Muhammad Khalid Abdullah:** Resources, Investigation. **Ahmad Zainul Hasan:** Resources, Data curation, Formal analysis, Validation. **Imam Asrowardi:** Software, Writing – review & editing. **Fitriani:** Resources, Writing – review & editing. **Muhammad Tahir:** Supervision, Conceptualization, Funding acquisition.

## Data Availability

FigshareUntargeted metabolomic datasets of various cloves species (Original data)
